# Di­aqua­tetra­kis­(1*H*-imidazole-κ*N* ^3^)magnesium dichloride

**DOI:** 10.1107/S1600536813021478

**Published:** 2013-08-10

**Authors:** M. Kayalvizhi, G. Vasuki, Kamel Kaabi, Cherif Ben Nasr

**Affiliations:** a Department of Physics, Kunthavai Naachiar Government Arts College (W) (Autonomous), Thanjavur-7, India; b Laboratoire de Chimie des Matériaux, Faculté des Sciences de Bizerte, 7021 Zarzouna, Tunisia

## Abstract

In the title compound, [Mg(C_3_H_3_N_2_)_4_(H_2_O)_2_]Cl_2_, the Mg^II^ cation lies on a crystallographic inversion centre and is coordinated by two water mol­ecules and four N-atom donors from monodentate imidazole ligands, giving a slightly distorted octa­hedral stereochemistry. In the crystal, water O—H⋯Cl and imidazole N—H⋯Cl hydrogen bonds give rise to a three-dimensional structure.

## Related literature

For a similar structure, see: Reiss *et al.* (2011 ▶).
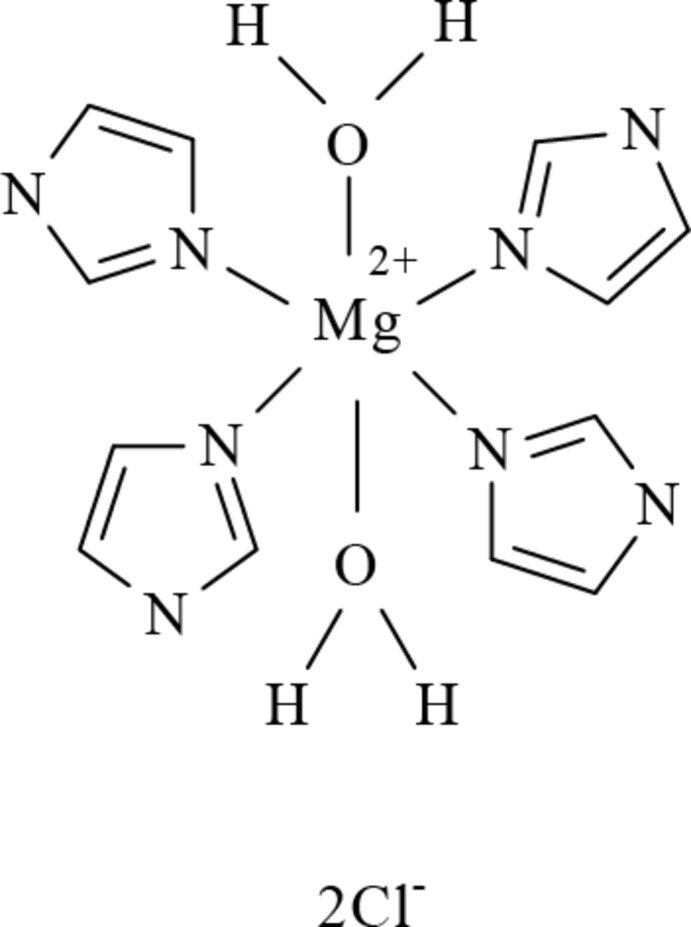


## Experimental

### 

#### Crystal data

[Mg(C_3_H_3_N_2_)_4_(H_2_O)_2_]Cl_2_
*M* *_r_* = 403.57
Monoclinic, 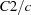

*a* = 12.3826 (6) Å
*b* = 11.0048 (4) Å
*c* = 14.4485 (6) Å
β = 107.037 (1)°
*V* = 1882.47 (14) Å^3^
*Z* = 4
Mo *K*α radiation
μ = 0.40 mm^−1^
*T* = 296 K
0.30 × 0.25 × 0.20 mm

#### Data collection

Bruker Kappa APEXII CCD diffractometer
Absorption correction: multi-scan (*SADABS*; Bruker, 1999 ▶) *T*_min_ = 0.889, *T*_max_ = 0.924
8496 measured reflections
1854 independent reflections
1695 reflections with *I* > 2σ(*I*)
*R*_int_ = 0.026

#### Refinement

*R*[*F* ^2^ > 2σ(*F* ^2^)] = 0.025
*wR*(*F* ^2^) = 0.068
*S* = 1.05
1854 reflections
132 parameters
4 restraints
H atoms treated by a mixture of independent and constrained refinement
Δρ_max_ = 0.17 e Å^−3^
Δρ_min_ = −0.25 e Å^−3^

### 

Data collection: *APEX2* (Bruker, 2004 ▶); cell refinement: *APEX2* and *SAINT* (Bruker, 2004 ▶); data reduction: *SAINT* and *XPREP* (Bruker, 2004 ▶); program(s) used to solve structure: *SHELXS97* (Sheldrick, 2008 ▶); program(s) used to refine structure: *SHELXL97* (Sheldrick, 2008 ▶); molecular graphics: *ORTEP-3 for Windows* (Farrugia, 2012 ▶); software used to prepare material for publication: *PLATON* (Spek, 2009 ▶).

## Supplementary Material

Crystal structure: contains datablock(s) I, global. DOI: 10.1107/S1600536813021478/zs2271sup1.cif

Structure factors: contains datablock(s) I. DOI: 10.1107/S1600536813021478/zs2271Isup2.hkl

Additional supplementary materials:  crystallographic information; 3D view; checkCIF report

## Figures and Tables

**Table 1:** Selected bond lengths (Å)

Mg1—N1	2.2281 (10)
Mg1—N3	2.1611 (10)
Mg1—O1	2.0923 (9)

**Table 2:** Hydrogen-bond geometry (Å, °)

*D*—H⋯*A*	*D*—H	H⋯*A*	*D*⋯*A*	*D*—H⋯*A*
O1—H1*W*⋯Cl1^i^	0.84 (1)	2.30 (1)	3.1361 (9)	172 (2)
O1—H2*W*⋯Cl1	0.84 (1)	2.30 (1)	3.1337 (10)	176 (2)
N2—H2*A*⋯Cl1^ii^	0.89 (1)	2.47 (1)	3.3165 (12)	160 (2)
N4—H4*A*⋯Cl1^iii^	0.89 (1)	2.43 (1)	3.2585 (13)	155 (2)

Symmetry codes: (i) 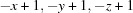; (ii) 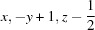; (iii) 
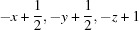
.

**Fig. 1.**
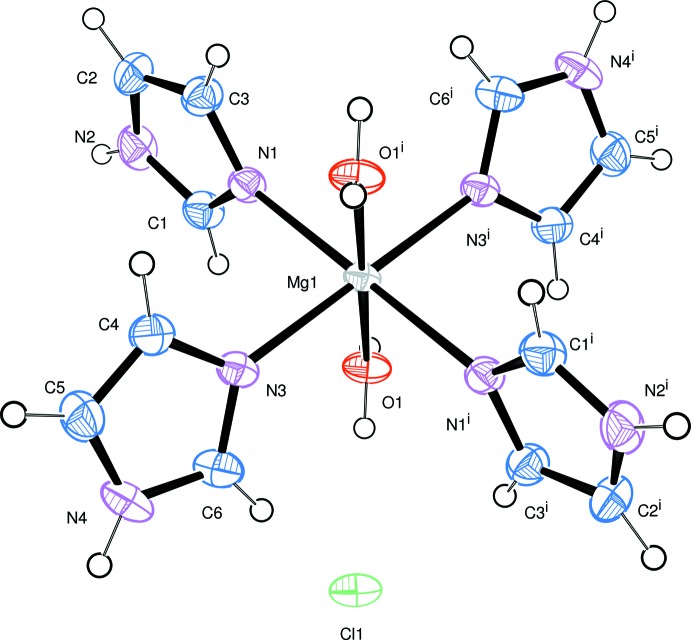
The molecular structure of the title compound showing atom numbering, with displacement ellipsoids drawn at the 40% probability level. For symmetry code (i): -x + 1/2, -y + 3/2, -z + 1.

**Fig. 2.**
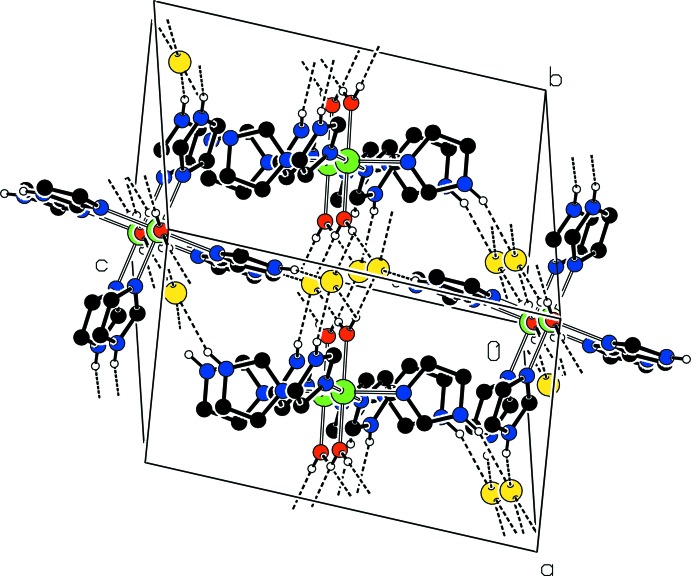
Crystal packing of the title compound viewed along the c axis. Dashed lines indicate hydrogen bonds. H atoms not involved in hydrogen bonding have been omitted for clarity.

## supplementary crystallographic information

### 1. Comment

In the title compound, [Mg(C_3_H_3_N_2_)_4_(H_2_O)_2_] . 2Cl, the Mg^II^ cation lies on a crystallographic inversion centre and is coordinated by two water molecules and four *N*-atom donors from monodentate imidazole ligands, (Fig. 1), giving a slightly distorted octahedral geometry (Table 1). In the crystal, O—H···Cl and N—H···Cl hydrogen bonds between both the aqua ligands and the imidazole ligands and the chloride counter-anions (Table 2) generate a three-dimensional structure (Fig. 2). These water–chloride hydrogen-bonding interactions are in the typical range as observed in the redetermined structure of diaquatetrakis(dimethylformamide-κ*O*)magnesium dichloride (Reiss *et al.*, 2011).

### 2. Experimental

A solution of MgCl_2_ (0.2 mmol) in water (6 ml) was added dropwise to a solution of imidazole (0.8 mmol) in ethanol. After stirring for 30 min, the mixture was filtered. Crystals suitable for X-ray analysis were obtained by evaporating the filtrate at room temperature (yield 56%).

### 3. Refinement

Carbon-bound H atoms were placed at calculated positions and treated as riding on the parent atom, with, C—H = 0.93 Å and with *U*_iso_(H) = 1.2*U*_eq_(C). The O-bound and N-bound H atoms were located in a difference Fourier map and refined freely.

### Crystal data

**Table:**

[Mg(C_3_H_4_N_2_)_4_(H_2_O)_2_]Cl_2_	*F*(000) = 840
*M**_r_* = 403.57	*D*_x_ = 1.424 Mg m^−^^3^
Monoclinic, *C*2/*c*	Mo *K*α radiation, λ = 0.71073 Å
Hall symbol: -C 2yc	Cell parameters from 8496 reflections
*a* = 12.3826 (6) Å	θ = 2.1–26.0°
*b* = 11.0048 (4) Å	µ = 0.40 mm^−^^1^
*c* = 14.4485 (6) Å	*T* = 296 K
β = 107.037 (1)°	Block, colourless
*V* = 1882.47 (14) Å^3^	0.30 × 0.25 × 0.20 mm
*Z* = 4	

### Data collection

**Table:**

Bruker Kappa APEXII CCD diffractometer	1854 independent reflections
Radiation source: fine-focus sealed tube	1695 reflections with *I* > 2σ(*I*)
Graphite monochromator	*R*_int_ = 0.026
ω and φ scan	θ_max_ = 26.0°, θ_min_ = 2.5°
Absorption correction: multi-scan (*SADABS*; Bruker, 1999)	*h* = −15→14
*T*_min_ = 0.889, *T*_max_ = 0.924	*k* = −13→13
8496 measured reflections	*l* = −17→16

### Refinement

**Table:**

Refinement on *F*^2^	Secondary atom site location: difference Fourier map
Least-squares matrix: full	Hydrogen site location: inferred from neighbouring sites
*R*[*F*^2^ > 2σ(*F*^2^)] = 0.025	H atoms treated by a mixture of independent and constrained refinement
*wR*(*F*^2^) = 0.068	*w* = 1/[σ^2^(*F*_o_^2^) + (0.0327*P*)^2^ + 1.0653*P*] where *P* = (*F*_o_^2^ + 2*F*_c_^2^)/3
*S* = 1.05	(Δ/σ)_max_ < 0.001
1854 reflections	Δρ_max_ = 0.17 e Å^−^^3^
132 parameters	Δρ_min_ = −0.25 e Å^−^^3^
4 restraints	Extinction correction: *SHELXL97* (Sheldrick, 2008), Fc^*^=kFc[1+0.001xFc^2^λ^3^/sin(2θ)]^-1/4^
Primary atom site location: structure-invariant direct methods	Extinction coefficient: 0.0093 (6)

### Special details

**Table:**

Geometry. All e.s.d.'s (except the e.s.d. in the dihedral angle between two l.s. planes) are estimated using the full covariance matrix. The cell e.s.d.'s are taken into account individually in the estimation of e.s.d.'s in distances, angles and torsion angles; correlations between e.s.d.'s in cell parameters are only used when they are defined by crystal symmetry. An approximate (isotropic) treatment of cell e.s.d.'s is used for estimating e.s.d.'s involving l.s. planes.
Refinement. Refinement of *F*^2^ against ALL reflections. The weighted *R*-factor *wR* and goodness of fit *S* are based on *F*^2^, conventional *R*-factors *R* are based on *F*, with *F* set to zero for negative *F*^2^. The threshold expression of *F*^2^ > σ(*F*^2^) is used only for calculating *R*-factors(gt) *etc*. and is not relevant to the choice of reflections for refinement. *R*-factors based on *F*^2^ are statistically about twice as large as those based on *F*, and *R*- factors based on ALL data will be even larger.

### Fractional atomic coordinates and isotropic or equivalent isotropic displacement parameters (Å^2^)

**Table:**

	*x*	*y*	*z*	*U*_iso_*/*U*_eq_	
C1	0.29321 (12)	0.73665 (13)	0.29376 (10)	0.0376 (3)	
H1	0.3423	0.6718	0.3154	0.045*	
C2	0.19291 (13)	0.87405 (14)	0.19885 (10)	0.0440 (4)	
H2	0.1594	0.9221	0.1451	0.053*	
C3	0.17954 (11)	0.88354 (12)	0.28794 (9)	0.0366 (3)	
H3	0.1340	0.9406	0.3059	0.044*	
C4	0.01955 (12)	0.63009 (13)	0.37687 (10)	0.0393 (3)	
H4	−0.0098	0.7052	0.3525	0.047*	
C5	−0.03394 (12)	0.52348 (14)	0.35343 (11)	0.0462 (4)	
H5	−0.1058	0.5111	0.3112	0.055*	
C6	0.13086 (11)	0.49423 (12)	0.45568 (11)	0.0385 (3)	
H6	0.1931	0.4549	0.4967	0.046*	
N1	0.24289 (8)	0.79672 (9)	0.34828 (7)	0.0298 (2)	
N2	0.26524 (11)	0.78024 (12)	0.20364 (8)	0.0434 (3)	
N3	0.12407 (9)	0.61208 (9)	0.44212 (7)	0.0298 (2)	
N4	0.03796 (11)	0.43755 (11)	0.40368 (10)	0.0429 (3)	
O1	0.37963 (8)	0.62540 (8)	0.50646 (7)	0.0355 (2)	
Mg1	0.2500	0.7500	0.5000	0.02385 (15)	
Cl1	0.41012 (3)	0.35283 (3)	0.57173 (3)	0.04135 (14)	
H1W	0.4370 (11)	0.6384 (16)	0.4884 (12)	0.057 (5)*	
H2A	0.2880 (16)	0.7492 (16)	0.1556 (10)	0.069 (6)*	
H2W	0.3849 (15)	0.5529 (10)	0.5253 (12)	0.056 (5)*	
H4A	0.0289 (15)	0.3576 (9)	0.4011 (13)	0.058 (5)*	

### Atomic displacement parameters (Å^2^)

**Table:**

	*U*^11^	*U*^22^	*U*^33^	*U*^12^	*U*^13^	*U*^23^
C1	0.0447 (7)	0.0340 (7)	0.0385 (7)	0.0014 (6)	0.0188 (6)	−0.0019 (6)
C2	0.0528 (8)	0.0486 (9)	0.0284 (7)	−0.0021 (7)	0.0084 (6)	0.0050 (6)
C3	0.0413 (7)	0.0359 (7)	0.0336 (7)	0.0034 (6)	0.0125 (6)	0.0017 (6)
C4	0.0389 (7)	0.0341 (7)	0.0413 (8)	−0.0016 (6)	0.0062 (6)	0.0048 (6)
C5	0.0416 (7)	0.0472 (9)	0.0460 (8)	−0.0129 (7)	0.0071 (6)	−0.0033 (7)
C6	0.0357 (7)	0.0278 (7)	0.0535 (8)	0.0005 (5)	0.0157 (6)	0.0041 (6)
N1	0.0356 (5)	0.0277 (5)	0.0288 (5)	−0.0026 (4)	0.0138 (4)	−0.0002 (4)
N2	0.0558 (7)	0.0484 (7)	0.0325 (6)	−0.0074 (6)	0.0233 (5)	−0.0088 (5)
N3	0.0326 (5)	0.0251 (5)	0.0337 (6)	−0.0023 (4)	0.0130 (4)	0.0007 (4)
N4	0.0485 (7)	0.0261 (6)	0.0594 (8)	−0.0110 (5)	0.0244 (6)	−0.0065 (5)
O1	0.0360 (5)	0.0257 (5)	0.0523 (6)	0.0056 (4)	0.0248 (4)	0.0073 (4)
Mg1	0.0275 (3)	0.0196 (3)	0.0275 (3)	−0.0003 (2)	0.0126 (2)	0.0008 (2)
Cl1	0.0432 (2)	0.0314 (2)	0.0584 (2)	0.00885 (13)	0.02879 (17)	0.01331 (14)

### Geometric parameters (Å, º)

**Table:**

C1—N1	1.3166 (16)	C6—N3	1.3107 (17)
C1—N2	1.3347 (18)	C6—N4	1.3306 (19)
C1—H1	0.9300	C6—H6	0.9300
C2—C3	1.3491 (19)	Mg1—N1	2.2281 (10)
C2—N2	1.355 (2)	N2—H2A	0.890 (9)
C2—H2	0.9300	Mg1—N3	2.1611 (10)
C3—N1	1.3738 (17)	N4—H4A	0.887 (9)
C3—H3	0.9300	Mg1—O1	2.0923 (9)
C4—C5	1.341 (2)	O1—H1W	0.838 (9)
C4—N3	1.3741 (17)	O1—H2W	0.839 (9)
C4—H4	0.9300	Mg1—O1^i^	2.0923 (9)
C5—N4	1.355 (2)	Mg1—N3^i^	2.1612 (10)
C5—H5	0.9300	Mg1—N1^i^	2.2281 (10)
			
N1—C1—N2	111.72 (13)	C6—N3—C4	104.57 (11)
N1—C1—H1	124.1	C6—N3—Mg1	129.03 (9)
N2—C1—H1	124.1	C4—N3—Mg1	126.30 (9)
C3—C2—N2	105.92 (12)	C6—N4—C5	107.41 (12)
C3—C2—H2	127.0	C6—N4—H4A	124.5 (12)
N2—C2—H2	127.0	C5—N4—H4A	128.0 (12)
C2—C3—N1	110.17 (12)	Mg1—O1—H1W	125.7 (12)
C2—C3—H3	124.9	Mg1—O1—H2W	128.6 (12)
N1—C3—H3	124.9	H1W—O1—H2W	105.6 (17)
C5—C4—N3	110.08 (13)	O1^i^—Mg1—O1	179.999 (1)
C5—C4—H4	125.0	O1^i^—Mg1—N3	89.19 (4)
N3—C4—H4	125.0	O1—Mg1—N3	90.81 (4)
C4—C5—N4	106.08 (12)	O1^i^—Mg1—N3^i^	90.81 (4)
C4—C5—H5	127.0	O1—Mg1—N3^i^	89.19 (4)
N4—C5—H5	127.0	N3—Mg1—N3^i^	180.0
N3—C6—N4	111.86 (13)	O1^i^—Mg1—N1^i^	90.22 (4)
N3—C6—H6	124.1	O1—Mg1—N1^i^	89.78 (4)
N4—C6—H6	124.1	N3—Mg1—N1^i^	91.99 (4)
C1—N1—C3	104.59 (11)	N3^i^—Mg1—N1^i^	88.01 (4)
C1—N1—Mg1	125.70 (9)	O1^i^—Mg1—N1	89.78 (4)
C3—N1—Mg1	129.45 (8)	O1—Mg1—N1	90.22 (4)
C1—N2—C2	107.60 (12)	N3—Mg1—N1	88.01 (4)
C1—N2—H2A	124.8 (12)	N3^i^—Mg1—N1	91.99 (4)
C2—N2—H2A	127.5 (12)	N1^i^—Mg1—N1	180.0
			
N2—C2—C3—N1	0.07 (16)	C4—N3—Mg1—O1^i^	33.40 (11)
N3—C4—C5—N4	−0.57 (17)	C6—N3—Mg1—O1	29.08 (12)
N2—C1—N1—C3	−0.09 (15)	C4—N3—Mg1—O1	−146.60 (11)
N2—C1—N1—Mg1	174.52 (9)	C6—N3—Mg1—N1^i^	−60.73 (12)
C2—C3—N1—C1	0.01 (15)	C4—N3—Mg1—N1^i^	123.59 (11)
C2—C3—N1—Mg1	−174.32 (9)	C6—N3—Mg1—N1	119.27 (12)
N1—C1—N2—C2	0.13 (17)	C4—N3—Mg1—N1	−56.41 (11)
C3—C2—N2—C1	−0.12 (16)	C1—N1—Mg1—O1^i^	−168.58 (11)
N4—C6—N3—C4	−0.12 (16)	C3—N1—Mg1—O1^i^	4.66 (11)
N4—C6—N3—Mg1	−176.52 (9)	C1—N1—Mg1—O1	11.42 (11)
C5—C4—N3—C6	0.43 (16)	C3—N1—Mg1—O1	−175.34 (11)
C5—C4—N3—Mg1	176.96 (10)	C1—N1—Mg1—N3	−79.38 (11)
N3—C6—N4—C5	−0.23 (17)	C3—N1—Mg1—N3	93.85 (11)
C4—C5—N4—C6	0.48 (17)	C1—N1—Mg1—N3^i^	100.62 (11)
C6—N3—Mg1—O1^i^	−150.92 (12)	C3—N1—Mg1—N3^i^	−86.14 (11)

Symmetry code: (i) −*x*+1/2, −*y*+3/2, −*z*+1.

### Hydrogen-bond geometry (Å, º)

**Table:**

*D*—H···*A*	*D*—H	H···*A*	*D*···*A*	*D*—H···*A*
O1—H1*W*···Cl1^ii^	0.84 (1)	2.30 (1)	3.1361 (9)	172 (2)
O1—H2*W*···Cl1	0.84 (1)	2.30 (1)	3.1337 (10)	176 (2)
N2—H2*A*···Cl1^iii^	0.89 (1)	2.47 (1)	3.3165 (12)	160 (2)
N4—H4*A*···Cl1^iv^	0.89 (1)	2.43 (1)	3.2585 (13)	155 (2)

Symmetry codes: (ii) −*x*+1, −*y*+1, −*z*+1; (iii) *x*, −*y*+1, *z*−1/2; (iv) −*x*+1/2, −*y*+1/2, −*z*+1.
